# A Network Pharmacology-Based Study on the Mechanism of Dibutyl Phthalate of *Ocimum basilicum* L. against Alzheimer's Disease through the AKT/GSK-3*β* Pathway

**DOI:** 10.1155/2022/9494548

**Published:** 2022-12-24

**Authors:** Jimilihan Simayi, Bayinsang, Maimaitiming Nuermaimaiti, Sendaer Hailati, Mengyuan Han, Zulihuma Reheman, Ainiwaer Wumaier, Wenting Zhou

**Affiliations:** ^1^Department of Pharmacology, Xinjiang Medical University, 830011 Urumqi, Xinjiang, China; ^2^Department of Pharmacy, Urumqi Maternal and Child Health Care Hospital, 830011 Urumqi, Xinjiang, China

## Abstract

**Background:**

*Ocimum basilicum* L. (OBL) is mainly used to treat neurological diseases in China. The preliminary work of this group showed that OBL improves cognitive impairment in Alzheimer's disease (AD). However, the underlying pharmacological mechanism remains unclear.

**Methods:**

The components of OBL were compiled by literature search, and their active ingredients were screened by online database. The drug targets of OBL in the treatment of AD were predicted and analyzed using information derived from sources such as the SwissTargetPrediction tool. And through the network visual analysis function of Cytoscape software and protein-protein interaction analysis (PPI), the core targets of OBL treatment of AD are predicted. Furthermore, Gene Ontology (GO) and Kyoto Encyclopedia of Genes and Genomes (KEGG) were employed to analyze the related signaling pathways affected by OBL. Moreover, AutoDock software was used to assess the potential binding affinity between the core targets and the active compounds. Subsequently, *in vivo* experiment was conducted to verify the findings of network pharmacology.

**Results:**

A total of 35 active compounds and 188 targets of OBL were screened, of which 43 common targets were related to AD. The active compounds of 35 OBLs induced 118 GO and 78 KEGG. The results of PPI and network topology parameter analysis show that targets such as MAPK1, GSK3B, NR3C2, ESR1, and EGFR are known as the core targets for the treatment of AD by OBL and are docked with the active ingredients of OBL. Molecular docking results suggest that diterbutyl phthalate (DBP) may be the main active component of OBL for the treatment of AD. Flow cytometry analysis results showed that apoptosis decreased with increasing DBP dose. In addition, DBP significantly decreased the levels of lactate dehydrogenase (LDH) and reactive oxygen species (ROS) in the supernatant of A*β*_25-35_-induced injury HT22 cell cultures, and it can be speculated that DBP has the ability to protect the stability of injured neuronal cells and improve the permeability of cell membranes, thus stabilizing the intracellular environment. Mechanistically, DBP may increase the mRNA levels of AKT, GSK-3*β*, etc. in AD cell models and regulate the phosphorylation of AKT/GSK-3*β* pathway-related.

**Conclusions:**

Conclusively, our study suggests that DBP, the main active component of OBL, has potential in the prevention or treatment of AD.

## 1. Introduction

Alzheimer's disease (AD) is a neurodegenerative disease characterized by amyloid protein (A) deposition and neurogenic fiber tangles [1]. Data shows that the number of people over 65 years of age with AD is expected to reach 6.7 million in 2025 [2]. Patients with AD present with symptoms such as short-term memory loss, language difficulties, and disorientation [3].There are many different theories on the pathogenesis of AD, such as the A cascade hypothesis, genetic mechanisms, inflammatory mechanisms, mitochondrial dysfunction, neurotransmitter dysregulation, glycogen synthase kinase-3 (GSK-3), and oxidative stress, but the pathogenesis has not been fully elucidated [4]. Research on AD is very limited, and active exploration of effective therapeutic drugs for AD has become an important research direction for therapeutic targets in recent years.

*Ocimum basilicum* L. (OBL), a genus of basil in the family Labiatae, has pharmacological effects such as free radical scavenging, anti-inflammatory, antitumor, antibacterial, hypolipidemic, and antiatherosclerotic [5]. OBL has been reported to have therapeutic activity against neurological disorders such as depression, anxiety, and sedation, mainly through anti-inflammatory and antioxidant properties [6]. Studies have reported the ameliorative effect of OBL volatile oil on neurodegenerative changes in mice caused by chronic unpredictable mild stress (CUMS), but the therapeutic effect of OBL on AD has not been studied and reported [7]. OBL ameliorates memory and neurological deficits after ischemia-reperfusion-induced brain injury in mice [6]. The composition of OBL is complicated and variable, and further research is needed to screen for active compounds with therapeutic effects, as well as to understand the role and mechanism of the compound in the treatment of AD.

**Figure 1:**
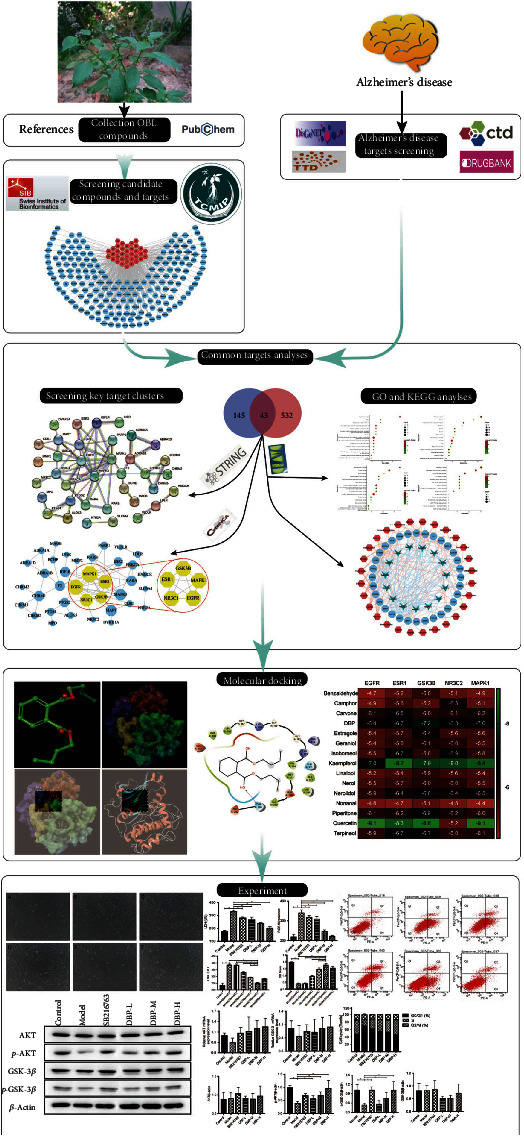
Workflow of the network pharmacological investigation on the use of DBP in AD treatment.

In this study, we investigated the active components, potential targets, and signaling pathways of OBL for the treatment of AD through network pharmacology, which is consistent with the therapeutic principles of Chinese medicine and ethnic medicine for the treatment of complex diseases and then searched for the best match between OBL small molecule compounds and target proteins by complementing the receptor active sites of AD disease targets with spatial structure and minimizing the binding energy. The monomeric component diterbutyl phthalate (DBP) was screened from OBL. *In vitro* experiments showed the effect of DBP on HT22 cells with A*β*_25-35_-induced damage. The material basis of the action of OBL and its mechanism of action was investigated to provide some theoretical basis for the treatment of clinical AD at a later stage, see Figure 1.

## 2. Materials and Methods

### 2.1. Collection and Collation of OBL Components

The OBL chemical components were collected according to the following criteria: (1) regional OBL component that has been publicly reported in the literature within the last 5 years and (2) chemical components that have been quantified in OBL. The collected components were preprocessed and standardized to remove outlier samples and redundant molecular descriptions, and finally, the component names were entered into the PubChem database (https://www.chemicalbook.com/) to retrieve normalized 3D molecular structure descriptors for subsequent data analysis.

### 2.2. Screening of OBL Candidate Components and Their Related Targets

The OBL-normalized 3D molecular structure descriptors were compiled according to the above criteria and entered into the SIB database (http://www.swissadme.ch/index.php) to derive pharmacokinetic parameters related to the chemical composition, and the biologically active components were selected for further study based on ADME parameters. In SwissADME, the high gastrointestinal absorption is indicated by the OB. The high OB value indicates the important index of pharmacodynamic molecules and drug-like properties. In this paper, high gastrointestinal absorption and bioavailability scores 0.55 were used as criteria for screening. The higher OB value is a key index indicating the potent molecules and drug-like properties. Pharmacodynamic effects can influence the ADME process and therefore lead to changes in drug bioavailability [8].

To estimate the drug similarity of each ingredient, pharmacokinetic parameters were calculated based on the model in the Pipeline Pilot ADMET collection. The obtained ingredients' English names were imported into the TCMIP V2.0 database (http://www.tcmip.cn/TCMIP/index.php) of herbal ingredients for searching to obtain the level of drug-likeness weight [9]. A quantitative index called the quantitative estimate of drug-likeness (QED) was used to assess drug similarity, and the estimated values ranged from 0 to 1. The mean QED values for drug-likeness were 0.49 and 0.67. If QED > 0.67 indicates good drug-likeness, 0.49 ≤ QED ≤ 0.67 indicates if QED > 0.67, it means that the drug forming property is good, 0.49 ≤ QED ≤ 0.67 means that the drug forming property is medium, and QED > 0.67 means that the drug forming property is weak. In this paper, 0.49 ≤ QED ≤ 0.67 was used as the screening criterion for screening. The achieved components were considered as candidate components of OBL, and the candidate components were collated through the SwissTargetPrediction (http://www.swisstargetprediction.ch/) database for their targets of action.

### 2.3. Screening of AD Disease Targets

Using “Alzheimer disease” as a keyword, we searched and screened known disease targets in four major disease-related databases, including DisGeNET (http://www.disgenet.org/), CTD (http://ctdbase.org/), TTD (https://db.idrblab.org/ttd/), and DrugBank (https://www.drugbank.ca), and eliminated repeated targets to obtain known targets for the pathogenesis of AD.

### 2.4. Drug-Disease Target Association Analysis

The OBL component targets were intersected with AD disease targets, and the intersected targets were imported into the STRING database to obtain the interaction relationship between the targets. With “homo sapiens” selected as the species, “minimum required interaction score” was selected as ≥0.7, and the rest of the default parameters were used. A protein-protein interaction (PPI) association network was constructed, and isolated nodes were deleted to obtain the initial network. The CytoNCA (2.1.6) plug-in was then used to determine the betweenness centrality (BC), closeness centrality (CC), eigenvector centrality (EC), degree centrality (DC), local average connectivity-based method (LAC), network centrality (NC), and other topological attributes as criteria for 2-step screening to simplify the network, screening different target clusters, and the top-ranked target clusters as key target clusters for OBL treatment of AD.

### 2.5. Gene Enrichment Analysis

In order to systematically elucidate the role of OBL in the treatment of AD, the intersecting targets of OBL for AD were subjected to GO (Gene Ontology) enrichment analysis and KEGG (Kyoto Encyclopedia of Genes and Genomes) signaling pathway enrichment analysis. The active component-target-pathway network map was constructed by Cytoscape 3.7.1 software (version: 3.7.1, https://cytoscape.org).

### 2.6. Pretreatment and Molecular Docking of Receptors and Ligands

#### 2.6.1. Ligand Pretreatment

The structures of the active ingredients obtained under 2.2 were corroborated with the help of the PubChem database and saved in mol format files. After checking the spatial structure in PyMOL software (https://pymol.org/), the structure was converted to PDB format. The structure is filled with AutoDock Tools 1.5.6, atomic charges are added, atomic types are assigned, and all flexible bonds are rotatable by default and saved in pdbqt format as docking ligands.

#### 2.6.2. Pretreatment of Receptors

The crystal structure proteins of the core targets of OBL therapeutic AD collected under 2.4 above were preprocessed using AutoTools, removing excess protein chains and ligands, hydrogenating to delete water molecules, calculating Gasteiger charges, and saving them as pdbqt files, which were used as receptors for molecular docking.

#### 2.6.3. Molecular Docking

The above pretreated ligands and receptors were docked to the proteins using AutoDock Vina (version: 1.2, http://vina.scripps.edu/index.html) for small molecules. Finally, the dominant conformation was taken for analysis and plotted using Maestro (Schrodinger) (https://www.schrodinger.com) software.

### 2.7. Cellular Experimental Validation

#### 2.7.1. Experimental Cell Lines

Mouse hippocampal neuronal cell HT22 cell line was obtained from Fenghui Bio.

#### 2.7.2. Drugs, Reagents, and Instruments

DBP (Ref. D806672: Maclean's); Reagents include fetal bovine serum (Ref. FND500: Excell Bio) and DMEM high sugar medium (Ref. C11965500BT: GIBCO). Instruments include the following: biological safety cabinet (Ref. HF1200LC, Shanghai Likang Instruments Co., Ltd.); CO_2_ cell incubator (Ref. Model: Smart Cell HF-90, Shanghai Likang Instruments Co., Ltd.); benchtop low-speed centrifuge (Model: DK-80, Shanghai Likang Instruments Co., Ltd.); PCR instrument (Model: ABI QuantStudio™ 6 Flex Real-Time PCR System, ABI); and enzyme marker (Model: xMarkTM, Bio-Rad).

#### 2.7.3. CCK-8 Method for the Detection of Low-, Medium-, and High-Dose Groups of DBP

A previous study by our group showed that A*β*_25-35_-inhibited HT22 cell survived in a concentration-dependent manner, with an IC_50_ of 173.568 *μ*m [10]. HT22 cells in good growth condition were digested with trypsin, prepared into 5 × 10^4^ cells/mL single cell suspension with complete medium, inoculated into 96-well plates (100 *μ*L/well, i.e., 5 × 10^3^ cells/well), incubated for 24 h at 37°C with 5% CO_2_ for wall attachment, the medium was discarded, and 100 *μ*L of A*β*_25-35_ at a final concentration of 173.568 *μ*mol/L (IC_50_) was added separately. L (IC_50_) of A*β*_25-35_ and different concentrations of DBP (0, 1, 5, 10, 20, and 50 *μ*mol) were added, while a blank control group was set up. After 48 h of intervention, the supernatant was collected for LDH assay, while 100 *μ*L of the configured 10% CCK-8 solution was added to each well, and the incubation was continued in the incubator for 1 h. After 1 h, the OD value at 450 nm was measured by enzyme marker. OD value at 450 nm was measured by ELISA after 1 h. The results of CCK-8 and LDH assay were combined to screen the low, medium, and high intervention concentrations of DBP for subsequent experiments.

#### 2.7.4. LDH and ROS Detection

HT22 cells in good growth state were taken, cells were digested with trypsin, prepared into 5 × 10^4^ cells/mL single cell suspension with complete medium, inoculated into 96-well plates (100 *μ*L/well, i.e., 5 × 10^3^ cells/well) and 6-well plates (2 mL/well, i.e., 1 × 10^5^ cells/well), incubated at 37°C and 5% CO_2_ for 24 h for wall attachment, and discarded medium, and the interventions were performed according to experimental groups, with 3 replicates per group. After the intervention was completed, the medium was discarded and the cells were collected for LDH and ROS detection (fluorescence detection wavelength setting: optimal excitation wavelength 500 nm and optimal emission wavelength 525 nm).

#### 2.7.5. Cell Cycle Detection by Flow Cytometry

HT22 cells in good growth condition were digested with trypsin, prepared into 5 × 10^4^ cells/mL single cell suspension with complete medium, inoculated into 25 cm^2^ culture flasks, and incubated in a 37°C, saturated humidity, 5% CO_2_ cell incubator for 24 h. After the intervention according to 2.6.1 experimental grouping, the cells in each group were digested with trypsin, washed with 5 mL of PBS, and fixed overnight at 4°C. Once, resuspend the cells with 500 *μ*L of precooled PBS, add the cell suspension to 3.5 mL of precooled 80% ethanol, and fix overnight at 4°C. Centrifuge at 2000 rpm for 5 min to precipitate the cells. Carefully aspirate the supernatant to avoid aspirating the cells. Wash 2 times with precooled PBS and discard the clean supernatant. Add 500 *μ*L PI/RNase Staining Buffer to resuspend the cells and pass through a 200 mesh nylon sieve to make a single cell suspension, incubate for 30 min at 4°C, protected from light, detect red fluorescence at an excitation wavelength of 488 nm with a flow cytometer, and detect light scattering. Analysis software was used for cellular DNA content analysis and light scattering.

#### 2.7.6. Flow Cytometry Detection of Apoptotic Cells

HT22 cells in good growth condition were digested with trypsin, prepared into 5 × 10^4^ cells/mL single cell suspension with complete medium, inoculated into 25 cm^2^ culture flask, and incubated in 37°C, saturated humidity, 5% CO_2_ cell culture chamber for 24 h. After the intervention according to the experimental grouping in 2.6.1, the culture fluid in the cell flask was aspirated into the centrifuge tube after the intervention was completed (containing the cells that were washed twice with PBS), and the PBS was collected together into the centrifuge tube. The cells were digested by trypsin, transferred to the centrifuge tube, and centrifuged at 1000 rpm for 5 min, and the supernatant was discarded. Wash the cells twice with precooled PBS and discard the supernatant. Add 500 *μ*L of 1 × Binding Buffer to resuspend the cells and pass through a 200 mesh sieve to make a single cell suspension. Add 5 *μ*L Annexin V-PE and 10 *μ*L 7-AAD to each tube, mix gently, and leave for 5 min at 4°C protected from light. Flow cytometry assay was performed within 30 minutes.

#### 2.7.7. Experimental Grouping

The experimental groupings are as follows: model control group (173.568 *μ*mol/L of A*β*_25-35_ intervened in HT22 cells for 48 h); positive control group (173.568 *μ*mol/L of A*β*_25-35_ and 0.5 *μ*mol/L of SB216763 together intervened in HT22 cells for 48 h); low-dose group of DBP (173.568 *μ*mol/L of A*β*_25-35_ and 50 *μ*mol/L DBP cointervened in HT22 cells for 48 h); DBP medium-dose group (173.568 *μ*mol/L of A*β*_25-35_ and 100 *μ*mol/L DBP cointervened in HT22 cells for 48 h); and DBP high-dose group (173.568 *μ*mol/L of A*β*_25-35_ and 150 *μ*mol/L DBP cointervened HT22 cells for 48 h).

#### 2.7.8. Detection of AKT and GSK-3*β* Gene Expression by qRT-PCR

HT22 cells in good growth condition were digested with trypsin and prepared into 5 × 10^4^ cells/mL single cell suspension with complete medium, inoculated into 25 cm^2^ culture flasks and incubated in 37°C, saturated humidity, 5% CO_2_ cell culture incubator for 24 h. The intervention was carried out according to experimental groups, with 5 replicates in each group. After the intervention was completed, the medium was discarded and 1 mL of TRIzol was added to digest the cells so that TRIzol lay flat on the cell level, and the cell culture flasks were repeatedly shaken until the cells were digested down and loaded into 1.5 mL EP tubes. Follow-up experiments were performed according to the qRT-PCR lab report.

#### 2.7.9. WB Detection of AKT, p-AKT, GSK-3*β*, and p-GSK-3*β* Protein Expression

HT22 cells in good growth condition were digested with trypsin, prepared into 5 × 10^4^ cells/mL single cell suspension with complete medium, inoculated into 25 cm^2^ culture flasks, and incubated in 37°C, saturated humidity, 5% CO_2_ cell incubator for 24 h. The intervention was carried out according to experimental groups, with 3 replicates in each group. After the intervention was completed, the culture medium in the flask was discarded, 3 mL of sterile PBS buffer was added and repeatedly rinsed twice, the PBS buffer was discarded, the cells were digested with trypsin, and the operation method was the same as cell passaging. After centrifugation, the supernatant was discarded and the cell precipitate was left, and the cells were collected after washing with 5 mL of sterile PBS. The subsequent experiments were performed according to the Western Blot experiment report.

#### 2.7.10. Data Processing

All data were expressed as mean ± standard deviation ($\bar{x}\pm \text{SD}$), and each group's data was statistically analyzed using SPSS 19.0 software. If the data conformed to normal distribution, one-way ANOVA was used for statistics: for Chi-squared, LSD method was used for multiple comparisons; for Chi-squared, Tamhane's method was used for multiple comparisons, and *P* < 0.05 indicated significant differences; if the data did not conform to normal distribution, the data were first log transformed to normalize the data, and then, the data were statistically analyzed according to the above one-way ANOVA method.

## 3. Results

### 3.1. Screening of Candidate Components for OBL

According to the composition collection criteria, 310 OBL components were compiled from published literature reports [5, 11–19], and these components were further investigated. Screening parameters of gastrointestinal absorption = high and bioavailability ≥ 0.55 were used to screen 107 ingredients, and then, 35 candidate ingredients were screened using 0.49 ≤ QED ≤ 0.67, as shown in Table 1.

### 3.2. Core Target Screening for OBL for AD

Canonical SMILES of the 35 candidate components obtained above were imported into the SwissTargetPrediction database to collect their action targets, and 525 action targets were obtained, and 188 action targets were obtained after deleting duplicate targets. The targets were linked to OBL components, and the component-target network map was constructed by Cytoscape software. The network consists of 223 nodes and 437 edges. The edges between the components (red octagonal shape) and the targets (blue circles) represent interactions.

**Figure 2:**
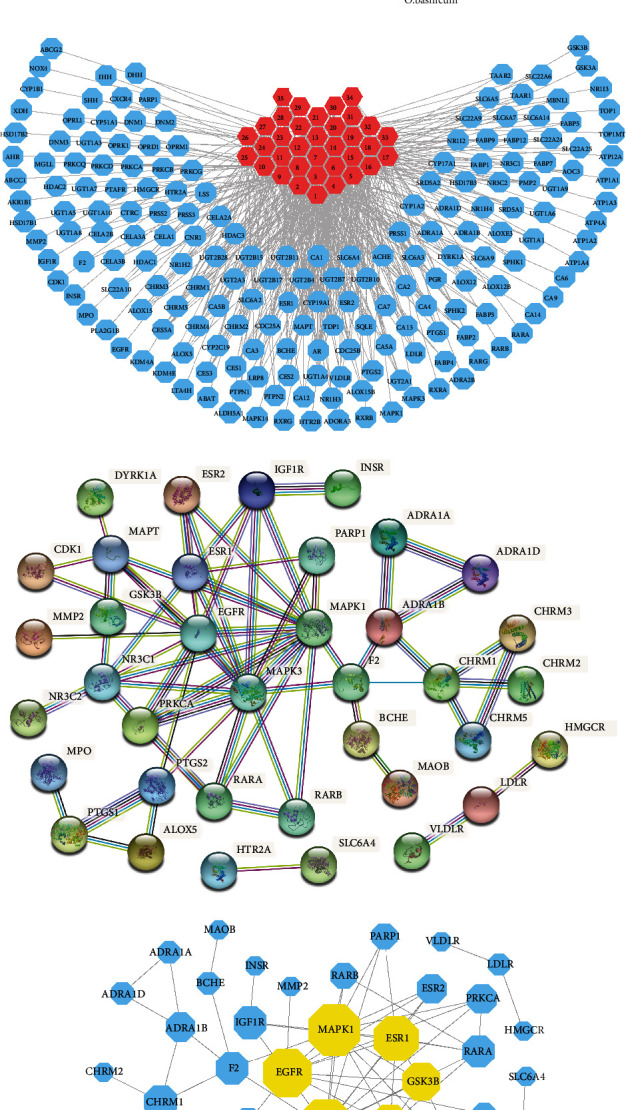
Core target screening for OBL for AD. (a) Combination of Venn diagram: 43 candidate targets were screened with the minimum required interaction score ≥ 0.7; (b) component-target network, including 223 nodes and 525 edges, the blue circles represent 188 candidate targets, the red octagonal shapes represent 35 OBL components; (c) PPI network diagram: including 37 nodes and 69 edges. (d) The core target of OBL in the treatment of AD.

The TTD, CTD, DisGeNET, and DrugBank databases were searched for targets related to AD pathogenesis to obtain 155, 298, 252, and 45 disease targets, respectively, and intersected with 188 OBL targets to obtain 43 shared targets, see Figure 2(a). STRING data screened 43 drug-disease shared targets with the criteria of “Homo sapiens” and ^“^minimum required interaction score^”^ ≥ 0.7. The 43 drug-disease targets were filtered by STRING data with the criteria of “Homo sapiens” and ^“^minimum required interaction score^”^ ≥ 0.7 to obtain 37 candidate targets, and the interaction relationship between the targets is shown in Figure 2(b). The network of 37 nodes and 69 edges was simplified as shown in Figure 2(c). The 46 candidate targets were filtered by the CytoNCA plug-in with the median of BC, CC, EC, DC, LAC, NC, and other topological attributes of the initial network nodes ≥ 2 and “value = Default” and “filter = used”. and “filter = used by total rank selected to 15% proteins” as filtering criteria to simplify the network and obtain mitogen-activated protein kinase 1 (MAPK1), glycogen synthase-3 beta (GSK3B), mineralocorticoid receptor (NR3C2), estrogen receptor (ESR1), and epidermal growth factor receptor (EGFR). The above five targets were used as core targets for molecular docking with OBL candidate components, see Figure 2(d) and Table 2.

### 3.3. GO and KEGG Enrichment Analysis Results

The results of GO gene function analysis of 43 common targets of OBL for AD treatment by the DAVID database showed that 289 entries were obtained, and the top 45 results were ranked in accordance with the corrected FDR pairs.

**Figure 3:**
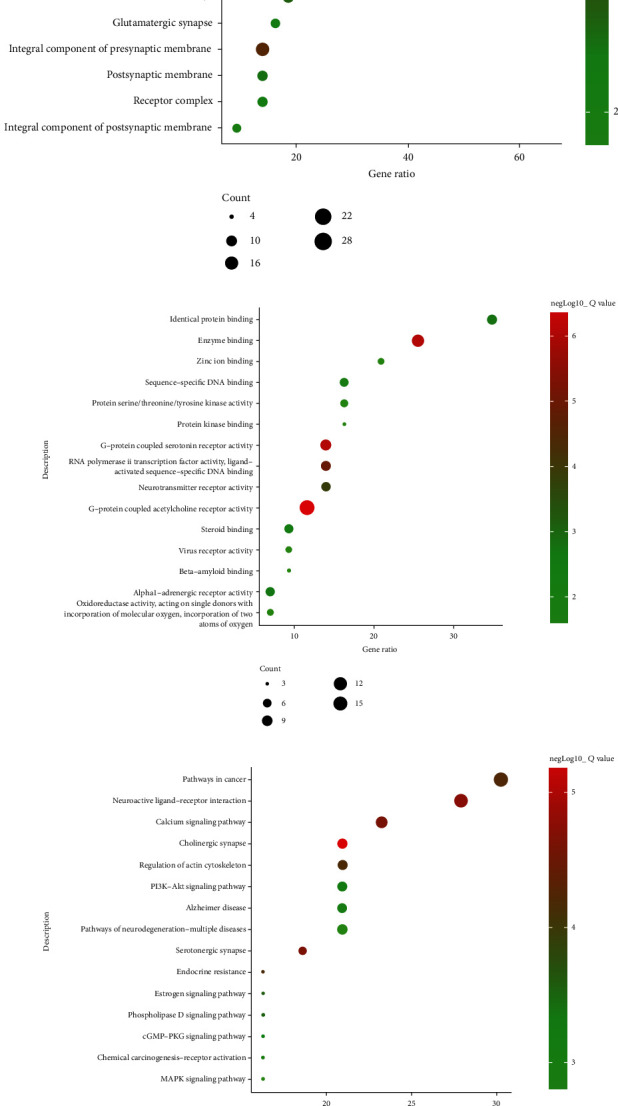
Analysis of gene work enrichment in the treatment of AD with OBL. (a) The first 15 biological processes. (b) Composition of the first 15 cells. (c) The first 15 molecular functions. (d) The first 15 signaling pathways. (e) C-T-P network diagram: red octagons represent 28 components, blue circles represent 32 candidate targets, and green triangles represent 15 signaling pathways.

G-protein-coupled acetylcholine receptor activity, enzyme binding, G-protein-coupled serotonin receptor activity, RNA polymerase II transcription factor activity, ligand-activated sequence-specific DNA binding, neurotransmitter receptor activity, identical protein binding, alpha1-adrenergic receptor activity, steroid binding, sequence-specific DNA binding, protein serine/threonine/tyrosine kinase activity, zinc ion binding, oxidoreductase activity, acting on single donors with incorporation of molecular oxygen, incorporation of two atoms of oxygen, virus receptor activity, protein kinase binding, and beta-amyloid binding are the first 15 biological functions induced by the 35 OBL components, see Figure 3(a).

Cytosol, nucleoplasm, nuclear chromatin, receptor complex, perinuclear region of cytoplasm, cytoplasm, cyclin-dependent protein kinase holoenzyme complex, transcription factor complex, plasma membrane, caveola, plasma membrane, integral component of plasma membrane, integral component of presynaptic membrane, dendrite, synapse, axon, postsynaptic membrane, macromolecular complex, receptor complex, glutamatergic synapse, integral component of postsynaptic membrane, membrane, nucleus, and mitochondrion were ranked among the top 15 cell components, see Figure 3(b).

The first 15 biological processes are adenylate cyclase-inhibiting G-protein-coupled acetylcholine receptor signaling pathway, G-protein-coupled serotonin receptor signaling pathway, signal transduction, positive regulation of vasoconstriction, G-protein-coupled acetylcholine receptor signaling pathway, peptidyl-tyrosine autophosphorylation, aging, G-protein-coupled receptor signaling pathway, coupled to cyclic nucleotide second messenger, response to xenobiotic stimulus, cellular response to reactive oxygen species, memory, intracellular steroid hormone receptor signaling pathway, cellular response to estradiol stimulus, adenylate cyclase-activating adrenergic receptor signaling pathway, and phospholipase C-activating G-protein-coupled receptor signaling pathway, see Figure 3(c).

Forty-three targets were imported into the DAVID database to analyze KEGG signaling pathways, and the pathways were further screened according to FDR size, with the FDR value indicating that the smaller the value in the enrichment analysis, the higher the enrichment significance. A total of 83 signaling pathways were collected and sorted according to FDR ≤ 0.05, and the top 15 results are shown in Figure 3, including pathways in cancer (hsa05200), neuroactive ligand-receptor interaction (hsa04080), calcium signaling pathway (hsa04020), cholinergic synapse (hsa04725), regulation of actin cytoskeleton (hsa04810), PI3K-AKT signaling pathway (hsa04151), Alzheimer's disease (hsa05010), pathways of neurodegeneration-multiple diseases (hsa05022), serotonergic synapse (hsa04726), endocrine resistance (hsa01522), estrogen signaling pathway (hsa04915), phospholipase D signaling pathway (hsa04072), chemical carcinogenesis-receptor activation (hsa05207), MAPK signaling pathway (hsa04010), and cGMP-PKG signaling pathway (hsa04022), see Figure 3(d).

Fifteen signaling pathway connecting targets and their acting OBL candidate components were used to construct a component-target-pathway (C-T-P) network map by Cytoscape software, which includes 75 nodes (including 28 candidate components, 32 candidate targets, and 15 signaling pathways) and 240 edges. The edges between components (red octagons), targets (blue circles), and signaling pathways (green triangles) represent interactions, see Figure 3(e).

### 3.4. Preliminary Validation of OBL Active Ingredient Action on AD Core Targets

To further determine the accuracy of the prediction results, molecular docking of the core targets to the candidate components was performed. The core targets EGFR (PDBID=5GTY), ESR1 (PDBID=4XI3), MAPK1 (PDBID=1TVO), GSK3B (PDBID=3mvh), and NR3C2 (PDBID=3VHU) and 35 candidate components were molecularly docked.

**Figure 4:**
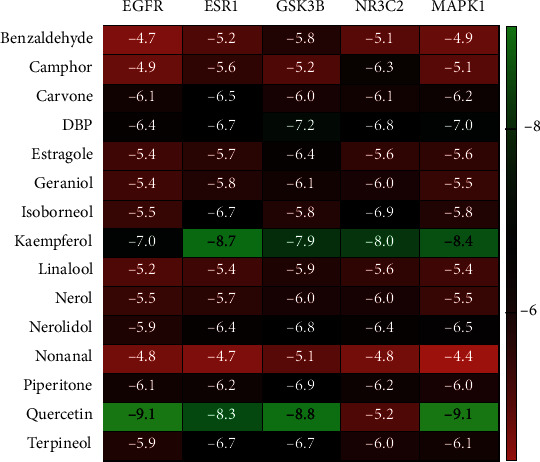
Docking results (BE ≤ −4.25 Kcal/mol): docking of 15 components with 5 core targets. The greener the color, the stronger the binding activity of the receptor to the ligand.

The magnitude of the binding energy (BE) is used to determine how well the components of OBL match the core target. When the conformation of ligand and receptor is stable, the lower the energy, the higher the possibility of action. Generally, BE ≤ −4.25 Kcal/mol indicates that the active ingredient has a certain binding energy to the target, BE ≤ −5.00 Kcal/mol indicates that the active ingredient has good binding energy to the target, and BE ≤ −7.00 Kcal/mol indicates that the active ingredient has strong binding energy. In this paper, using BE ≤ −4.25 Kcal/mol as the criterion, 15 of the 35 candidate components had some binding activity with 5 core targets, as shown in Table 3 and Figure 4. As can be seen in the table, EGFR was associated with Kaempferol (BE = −7) and Quercetin (BE = −9.1); ESR1 was associated with Kaempferol (BE = −8.7) and Quercetin (BE = −8.3); GSK3B with DBP (BE = −7.2), Kaempferol (BE = −7.9) and Quercetin (BE = −8.8), respectively; MAPK1 with Kaempferol (BE = −8.4) and Quercetin (BE = −9.1), respectively, and NR3C2 with Kaempferol (BE = −8) were less than -7.00 Kcal/mol, indicating a strong binding activity.

Quercetin can bind to GSK3B by patterning hydrophobic interactions with adjacent residues ASP292, THR291, THR211, LYS179, ALA177, MET227, GLU228, TYR229, ALA230, PHE438, MET281, LEU156, GLY157, GLY159, VAL164, GLU234, and ARG4, form hydrophobic interactions, and form hydrogen bonds with ALA230, thus binding to GSK3B. Quercetin binds to MAPK1 (ARG67, ILE56, LYS54, ALA52, GLY34, ILE31, LYS114, ASP111, MET108, LEU107, and LEU156). Quercetin can be activated at the active sites of the adjacent residues LEU844, THR854, ASP855, MET766, LEU777, LEU788, and ILE789. Quercetin can bind to EGFR by patterning hydrophobic interactions with adjacent residues LEU844, THR854, ASP855, MET766, LEU777, LEU788, ILE789, THR790, LEU792, MET793, GLY796, CYS797, LEU718, LEU1001, PHE997, VAL726, LYS745, ILE744, and ALA743 and *π*-interactions with LYS745 by patterning hydrophobic interactions with adjacent residues LEU428, TRP383, LEU384, LEU387, MET388, PHE404, LEU391, ARG394, GLU353, ALA350, LEU349, THR347, LEU346, MET343, MET528, LEU525, and VAL534 interactions, forming *π*-interactions with PHE404 and forming hydrogen bonds with ARG394, thus binding to ESR1.

Kaempferol can bind to EGFR by patterning hydrophobic interactions with adjacent residues ARG841, LEU844, THR790, ASP855, THR854, LYS745, ALA743, CYS797, GLY796, LEU718, GLY719, SER720, GLY721, GLY724, THR725, and VAL726. Kaempferol binds to ESR1 (MET140, ILE424, PHE425, MET343, LEU346, THR347, LEU349, ALA350, PHE404, ARG394, MET388, and LEU387). Kaempferol can be used as the active site of the residues MET281, THR291, ASP292, VAL164, LYS163, GLY162, GLY159, LYS158, GLY157, LEU156, ARG4, PHE442, PHE438, GLU234, LYS179, ILE180, and LEU181 forming hydrophobic interactions and thus binding to GSK3B. Kaempferol can bind to GSK3B by patterning hydrophobic interactions with adjacent residues LEU814, SER811, LEU810, MET852, LEU938, MET807, PHE941, CYS942, PHE956, THR945, LEU766, LEU769, MET845, ASN770, PHE829, LEU772, ALA773, and GLN776 forming hydrophobic interactions and thus binding to NR3C2. Kaempferol can bind to NR3C2 by forming hydrophobic interactions with adjacent residues MET108, ASP111, LEU156, ASN154, LYS114, SER153, ILE31, LYS151, GLU33, GLY34, CYS166, ASP167, YS54, GLY37, and VAL39 and thus binds to MAPK1 by patterning hydrophobic interactions with adjacent residues MET108, ASP154, LYS114, SER153, ILE31, LYS151, GLU33, GLY34, CYS166, ASP167, YS54, GLY37, and VAL39.

DBP binds to GSK3B (THR291, ASP292, ARG4, VAL164, LYS163, GLY162, PHE161, GLY159, LYS158, GLY157, LEU156, LEU181, LYS179, ALA177, GLU234, PHE438, ALA230, TYR229, MET281, GLU228, MET227, and THR211) active sites, and hydrogen bonds are formed with THR291, further improving the interaction between the ligand and GSK3B protein.

Among the above active ingredients, Quercetin and Kaempferol have been reported in the treatment of AD. For example, Quercetin can improve cholinergic function and play a neuroprotective role in AD. The neuroprotective effects of Quercetin have multiple mechanisms, including inhibition of A*β* aggregation [20], inhibition of NFT formation, inhibition of amyloid precursor protein (APP) cleavage enzyme (BACE1) inhibition [21], and acetylcholinesterase (AChE) inhibition [22] to reduce oxidative stress in AD [23]. It plays a role in alleviating Alzheimer's disease in terms of oxidative stress and reactive oxygen species scavenging, as well as improving vascular dysfunction and inhibiting inflammation. In contrast, Kaempferol delays the loss of climbing ability and memory and reduces oxidative stress and acetylcholinesterase activity in AD Drosophila [24].

**Figure 5:**
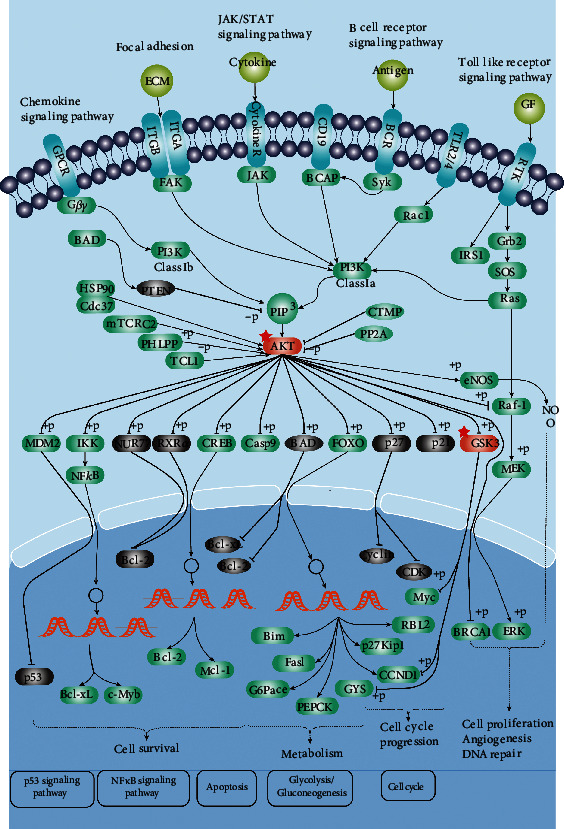
The PI3K/AKT signaling pathway (part). The red pentagram is the target of drug action.

**Figure 6:**
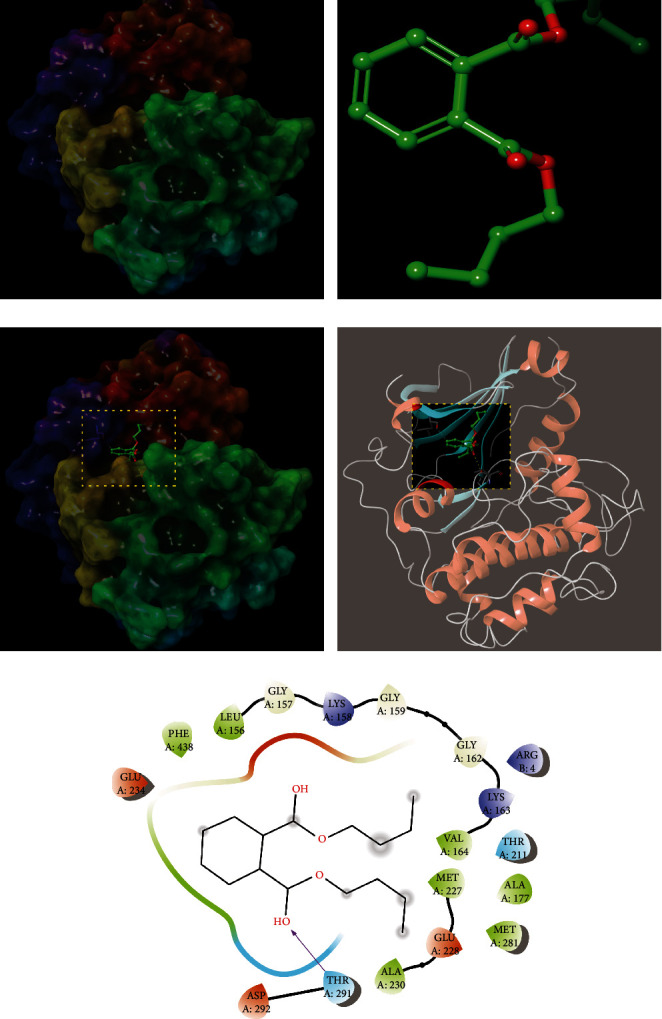
Molecular models of the binding of DBP to the predicted targets GSK3B shown as 3D diagrams and 2D diagrams.(a) 3D structure of GSK3B; (b) 3D structure of DBP; (c) DBP binding at the active site of GSK3B; (d) 3D pattern of DBP and GSK3B docking; (e) 3D pattern of DBP and GSK3B docking.

It was reported [25] that DBP could exacerbate hippocampal tissue damage in AD rats through oxidative stress and upregulate the Bcl-2/Bax/Caspase-3 signaling pathway, leading to decreased learning memory capacity. DBP exposure aggravates type 2 diabetes by disrupting the insulin-mediated PI3K/AKT signaling pathway [26], see Figure 5. DBP epigenetically induces reproductive toxicity via the PTEN/AKT pathway [27]. In this study, molecular docking results showed that DBP has strong binding activity to GSK3B, see Figure 6. Glycogen synthase kinase 3*β* (GSK-3B) is a key factor of the signal transduction pathway during oxidative stress in AD neurons [28]. A*β* in AD patients has neuronal toxicity and induces oxidative stress in neurons [29]. The downstream direct target gene of phosphatidylinositol (-3) kinase (PI3K) is GSK-3B, and A*β* is able to decrease AKT activity, increase GSK-3*β* activity, and inhibit AKT/GSK-3-related signaling pathways [30]. It can be seen that if GSK-3*β* can be effectively inhibited, it can help alleviate the symptoms of AD patients.

Researchers have shown that inhibitors of GSK-3*β* include Thiazolidinones (TZD), Bis-indole [31], Aniline [32], Maleimides, Kenpaullone [33], and Indirubin [34], while little research has been done on the effects of phthalates on AD. The blood-brain barrier (BBB), resulting in low drug solubility and low bioavailability, has become a bottleneck in the current treatment of AD [35]. DBP has good blood-brain barrier permeability (BBB = 0.56) [36]. Whether DBP exerts its therapeutic effect on AD only by inhibiting AKT/GSK-3*β*, the next step is to conduct *in vitro* experiments to verify it, see Figure 5.

### 3.5. DBP Intervention Concentration Screening

**Figure 7:**
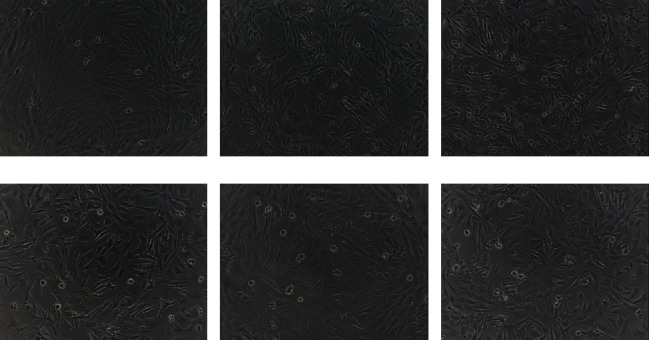
Effect of DBP on the HT22 AD model cell morphology after injured by A*β*_25-35_ (×100). (a) control group; (b) 170 *μ*mol/L A*β*_25-35_; (c) 50 *μ*mol/L DBP+170 *μ*mol/L A*β*_25-35_; (d) 100 *μ*mol/L DBP+170 *μ*mol/L A*β*_25-35_; (e) 150 *μ*mol/L DBP+170 *μ*mol/L A*β*_25-35_; (f) 200 *μ*mol/LDBP+170 *μ*mol/L A*β*_25-35_.

The OD values increased after different concentrations of DBP intervention in A*β*_25-35_-induced injury in the HT22 AD cell model of hippocampal neuronal cells in intervening mice. Compared with the model group, the OD values of DBP 50, 100, and 150 *μ*mol/L increased significantly with the concentration gradient, and 150 *μ*mol/L increased more significantly with statistically significant differences (*P* < 0.01). Therefore, 50, 100, and 150 *μ*mol/L were chosen for the next experiment. As shown in Figure 7, the morphological detection of the effect of DBP on the state of A*β*_25-35_-induced injury HT22 cells, the morphological observation showed that DBP could reduce the damage of A*β*_25-35_-induced injury HT22 cells.

**Figure 8:**
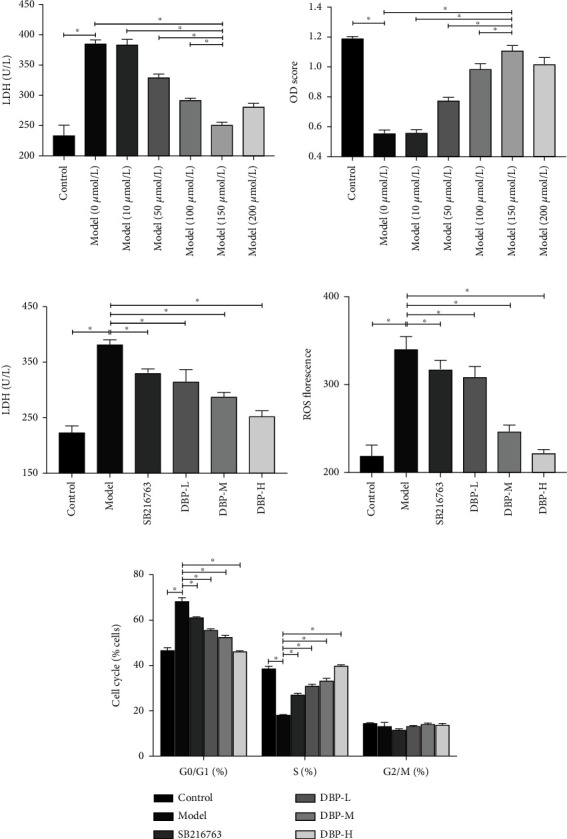
(a, b) The effect of A*β*_25-35_ on HT22 cell proliferation after treated with different concentration for 48 h. (c) Effects of different treatments on LDH expression level of HT22 cells. (d) ROS florescence. (e) Histograms of HT22 cell cycle in different intervention groups.

To further determine the effect of DBP on the activity of HT22 cells with A*β*_25-35_-induced injury, the LDH content in the cell supernatant was measured. The results showed a statistically significant decrease in LDL content with concentration gradient in the DBP 50, 100, and 150 *μ*mol/L groups compared with the model group (*P* < 0.01). In addition, the cell survival rate was significantly reduced after A*β*_25-35_ intervention in mouse hippocampal neuronal cells HT22. Based on the experimental results, 50, 100 *μ*mol/L, and 150 *μ*mol/L were selected as the low, medium, and high concentrations of DBP for subsequent experiments, see Figure 8.

### 3.6. LDH and ROS Assay Results

As shown in Figure 8(c), compared with the control group, the LDH content in the cell culture fluid of the DBP low-, medium-, and high-dose groups was significantly reduced, and the difference was statistically significant (*P* < 0.01). Compared with the model group, the LDH content in the cell cultures of the DBP low-, medium-, and high-dose groups was significantly lower than that of the model group, with a statistically significant difference compared with the model group (*P* < 0.01).

As shown in Figure 8(d), the fluorescence intensity of ROS in the cell culture medium of DBP low-, medium-, and high-dose groups was significantly reduced, and the difference was statistically significant (*P* < 0.01). Compared with the model group, the fluorescence intensity of ROS in the cell culture fluid of DBP low-, medium-, and high-dose groups was significantly reduced, and the difference was more significantly reduced in the high-dose group, and the difference was statistically significant (*P* < 0.01). This indicates that DBP can reduce oxidative stress and improve the viability of HT22 cells.

### 3.7. Cell Cycle Assay

The results are shown in Figure 8(e). Flow cytometric detection of DBP on the cell cycle after A*β*_25-35_-induced injury to HT22 cells showed that, compared with the model group, the A*β*_25-35_-induced injury to HT22AD cell model group showed a gradual decrease in the effect on the G0/G1 phase of the cell cycle with increasing DBP dose, a gradual increase in the effect on the S phase, and almost no effect on the G2/M phase.

### 3.8. Apoptosis Detection

**Figure 9:**
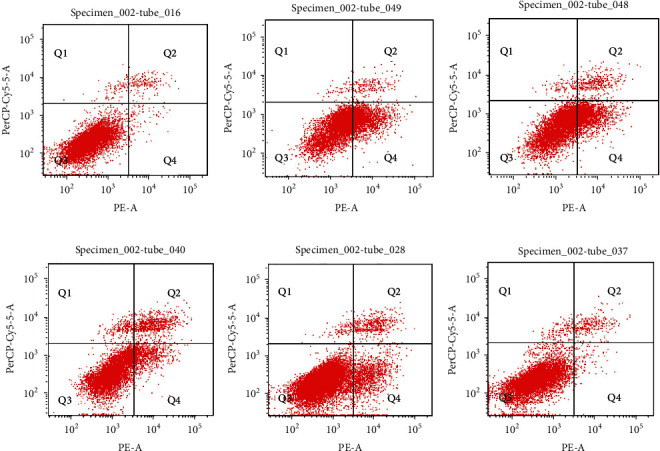
Effects of DBP on apoptosis of HT22 cells. (a) Control group; (b) Model group; (c) SB216763; (d) DBP-L; (e) DBP-M; (f) DBP-H.

**Figure 10:**
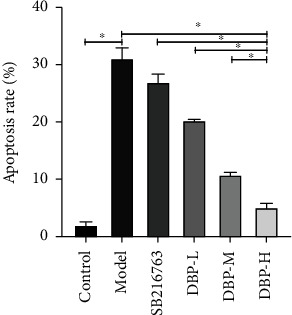
Effect of different concentrations of DBP intervention on apoptosis of HT22 cells.

Flow cytometry detected the apoptosis rate of A*β*_25-30_ after A*β*_25-35_ induced HT22 cell injury (Figures 9 and 10. The results showed that A*β*_25-35_ significantly promoted the apoptosis rate of HT22 cells (*P* < 0.01). After DBP intervention in the low-, medium-, and high-dose groups compared with the model group, A*β*_25-35_ produced damage to HT22 cells, and the apoptosis rate of damage gradually decreased with the increase of concentration. Among them, DBP was more obvious at medium and high doses, and the difference was statistically significant (*P* < 0.01) (Figure 10).

### 3.9. qRT-PCR Assay Results

**Figure 11:**
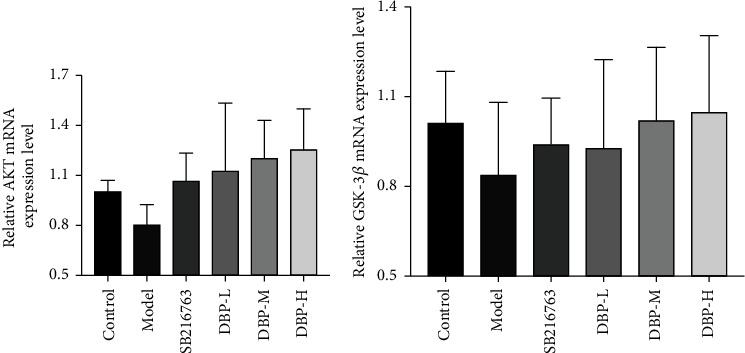
Gene expression level of HT22 cells in each group. Compared with the model group, AKT and GSK-3*β* mRNA expression level difference was not statistically significant (*P* > 0.05).

The results of reverse transcription quantitative PCR of AKT and GSK-3*β* mRNA expression after different intervention groups are shown in Figure 11. The effect of DBP on AKT and GSK-3*β* mRNA expression was increased in a concentration-dependent manner. Among them, compared with the model group, the AKT and GSK-3*β* mRNA expression levels of DBP low-, medium-, and high-dose groups increased with the gradient of DBP concentration, and the difference was not statistically significant.

### 3.10. WB Test Results

**Figure 12:**
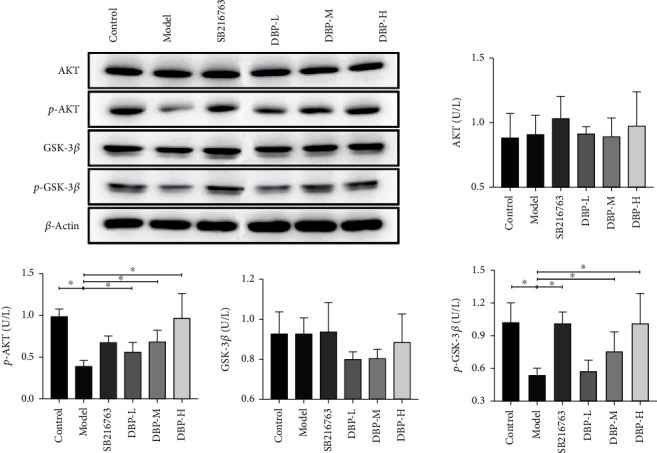
The western blot results of detecting the target proteins AKT, p-AKT, GSK-3*β*, and p-GSK-3*β* and the internal reference protein *β*-actin. Compared with model group, p-AKT and p-GSK-3*β* protein expressions were significantly increased (*P* < 0.05).

The WB results for detection of target protein AKT, p-AKT, GSK-3*β*, and p-GSK-3*β* and internal reference protein *β*-actin are identified separately in Figure 12. The sizes of the bands in the graphs match the sizes of the proteins. Among them, p-AKT and p-GSK-3*β* protein expression was significantly increased in a concentration-dependent manner in the DBP low-, medium-, and high-dose groups compared with the model group, and the expression levels were more pronounced in the high dose, with statistically significant differences (*P* < 0.05).

## 4. Discussion

Natural products have complex biological activities; their components are complex and diverse, and the composition of the formula is even more complex. In the field of TCM, natural products are commonly used in disease treatment, and their pathways and modes of action vary after entering the human body. They can act directly on specific targets, produce new products after metabolism, or act indirectly through the regulation of endogenous substances, exerting multicomponent, multitarget regulation. It is urgent to establish new research strategies and methods that can reflect the overall characteristics of TCM-ethnic medicine. In recent years, the rapid development of network pharmacology in TCM-ethnic medicine research has attracted attention, which integrates three aspects of TCM-ethnic medicine, including components, targets, and related diseases, and constructs a multidimensional network of “components-targets-pathways-diseases.” The active ingredients and mechanisms of action of TCM-ethnic medicine for diseases are then visualized and analyzed.

Visual analysis of the PPI network and its CytoNCA network revealed multiple associations between targets, with higher connectivity values associated with greater potential therapeutic effects. The top-ranked target clusters of MAPK1, EGFR, NR3C2, ESR1, and GSK3B may be key targets. In previous studies, MAPK1 has been associated in previous studies with neurodegeneration, synaptic plasticity, cell survival, and a role in autophagic vesicle formation in AD [37, 38]. The MAPK1 gene is thought to be an age-dependent transcriptional alteration gene involved in aberrant hyperphosphorylation of tau proteins, leading to aggregated neurogenic fiber tangles [39]. Furthermore, galantamine can treat Alzheimer's disease by attenuating the activation of MAPK1 [40].

EGFR is a transmembrane receptor with tyrosine kinase activity and is an important member of the ErbB family that is involved in regulating brain development, neuronal survival, and functional regulation, among other activities. Many neurodegenerative diseases include AD [41]. High levels of EGFR may improve the metabolism of pathological cerebrospinal fluid biomarkers associated with AD in cognitively normal middle-aged individuals [42]. Many recent studies have shown that EGFR inhibitors enhance autophagy, improve A*β* toxicity, and neuroinflammation [43].

NR3C2 is an important gene involved in the stress response, and its gene product, salt cortico­steroid receptor, is mainly distributed in the hippocampus and amygdala regions involved in the regulation of tension and anxiety and is closely related to tension and anxiety generation and regulation and cognitive function [44]. miR-135b-5p upregulation can reduce neuronal damage and inflammatory response in PSCI by targeting NR3C2, which is useful for poststroke cognitive impairment treatment [45].

Estrogen can cross the blood-brain barrier to act in the brain [46], and the action of estrogen is dependent on at least 2 ESRs (ESR1 and ESR2), potential candidate genes that regulate the development of AD. Variants in the ESR1 gene have been reported to regulate the susceptibility or course of AD. Scacchi et al. may be another gene that promotes interindividual variation in response to treatment with cholinesterase inhibitors (ChEIs) of genes [47].

KEGG enrichment pathway analysis revealed that neuroactive ligand-receptor interaction [48], PI3K-AKT signaling pathway [49], cholinergic synapse [50], regulation of actin cytoskeleton [51], Alzheimer's disease, pathways of neurodegeneration-multiple diseases, MAPK signaling pathway [52], and cGMP-PKG signaling pathway [53] are important pathways related to disease regulation in AD and have been reported in the literature, suggesting that the pathways predicted to be enriched in this study have high confidence. Quercetin mediates activation of the PI3K/AKT/GSK-3*β* signaling pathway through ER and has a protective effect against A*β*_25–35_-induced damage in PC12 cells [54]. Quercetin protects against okadaic acid- (OA-) induced hippocampal neuronal injury in HT22, a cell line derived from mouse hippocampal neurons, via MAPK and PI3K/AKT/GSK3*β* signaling pathways [55]. Kaempferol exposure delayed the loss of climbing ability, memory, and reduced oxidative stress and acetylcholinesterase activity in Drosophila AD [24].

The results of the present study showed that DBP was able to reduce the rate of inhibition of HT22 cells with A*β*_25–35_-induced damage, and the results indicated that low doses of DBP had a protective effect on HT22 cells with A*β*_25–35_-induced damage. The present study showed that DBP significantly reduced the LDH and ROS content in the supernatant of A*β*_25-35_-induced injury HT22 cell cultures, and DBP was also able to reduce the apoptosis rate of A*β*_25-35_-induced injury HT22 cells. Thus, it can be speculated from the results of this study that DBP has the ability to protect the stability of injured neuronal cells and improve the permeability of the cell membrane, thus stabilizing the intracellular environment. This effect may be related to the fact that DBP increases the mRNA levels of AKT, GSK-3*β*, etc. in AD cell models and regulates the phosphorylation of the AKT/GSK-3*β* pathway.

In summary, OBL has been used to explain the relationship between OBL active ingredients, potential targets, signaling pathways, and the pathogenesis of AD disease at a holistic level through network pharmacology technology and to verify the pharmacodynamic and regulatory mechanisms of OBL main active ingredients through *in vitro* experimental methods. This paper provides a new idea for the treatment of AD with complex pathogenesis and also lays the foundation for the in-depth study of the synergistic mechanism of OBL.

## Figures and Tables

**Table 1:** Screening of candidate components of OBL.

Compounds	Canonical SMILES	Gastrointestinal absorption	Lipinski violation	Bioavailability	Drug-likeness weight	Reference
Geraniol	CC(=CCCC(=CCO)C)C	High	0	0.55	0.617	[1, 4]
*β*-Citronellol	CC(CCC=C(C)C)CCO	High	0	0.55	0.606	[1]
Nerol	CC(=CCCC(=CCO)C)C	High	0	0.55	0.617	[1, 4]
Nerolidol	CC(=CCCC(=CCCC(C)(C=C)O)C)C	High	0	0.55	0.626	[1, 4, 5]
Linalool	CC(=CCCC(C)(C=C)O)C	High	0	0.55	0.617	[1, 4]
Nonanoic acid	CCCCCCCCC(=O)O	High	0	0.85	0.617	[1, 5]
P-coumaric acid	C1 = CC(=CC=C1C=CC(=O)O)O	High	0	0.85	0.654	[1]
Carvone	CC1 = CCC(CC1 = O)C(=C)C	High	0	0.55	0.525	[1]
Piperitone	CC1 = CC(=O)C(CC1)C(C)C	High	0	0.55	0.564	[1, 5]
*β*-Ionone	CC1 = C(C(CCC1)(C)C)C=CC(=O)C	High	0	0.55	0.613	[1, 5]
Estragole	COC1 = CC=C(C=C1)CC=C	High	0	0.55	0.599	[1, 2]
Quercetin	C1 = CC(=C(C=C1C2 = C(C(=O)C3 = C(C=C(C=C3O2)O)O)O)O)O	High	0	0.55	0.506	[1, 5]
Kaempferol	C1 = CC(=CC=C1C2 = C(C(=O)C3 = C(C=C(C=C3O2)O)O)O)O	High	0	0.55	0.637	[1, 5]
Norpseudoephedrine	CC(C(C1 = CC=CC=C1)O)N	High	0	0.55	0.656	[2]
Eucalyptol	CC1(C2CCC(O1)(CC2)C)C	High	0	0.55	0.519	[2, 4, 6]
Isoborneol	CC1(C2CCC1(C(C2)O)C)C	High	0	0.55	0.565	[2]
Bornyl acetate	CC(=O)OC1CC2CCC1(C2(C)C)C	High	0	0.55	0.634	[2, 4]
Methyleugenol	COC1 = C(C=C(C=C1)CC=C)OC	High	0	0.55	0.660	[2]
(-)-Norephedrine	CC(C(C1 = CC=CC=C1)O)N	High	0	0.55	0.656	[2]
Perilla alcohol	CC(=C)C1CCC(=CC1)CO	High	0	0.55	0.606	[3]
Camphor	CC1(C2CCC1(C(=O)C2)C)C	High	0	0.55	0.519	[4]
Terpineol	CC1 = CCC(CC1)C(C)(C)O	High	0	0.55	0.575	[4]
Caryophyllene oxide	CC1(CC2C1CCC3(C(O3)CCC2 = C)C)C	High	0	0.55	0.569	[4]
(-)-globulol	CC1CCC2C1C3C(C3(C)C)CCC2(C)O	High	0	0.55	0.667	[4]
(-)-spathulenol	CC1(C2C1C3C(CCC3(C)O)C(=C)CC2)C	High	0	0.55	0.622	[4]
*τ*-Cadinol	CC1 = CC2C(CCC(C2CC1)(C)O)C(C)C	High	0	0.55	0.531	[4]
*β*-Eudesmol	CC12CCCC(=C)C1CC(CC2)C(C)(C)O	High	0	0.55	0.670	[4]
*α*-Cadinol	CC1 = CC2C(CCC(C2CC1)(C)O)C(C)C	High	0	0.55	0.669	[4]
Neointermedeol	CC(=C)C1CCC2(CCCC(C2C1)(C)O)C	High	0	0.55	0.669	[4]
Decanoic acid	CCCCCCCCCC(=O)O	High	0	0.85	0.606	[4]
Isoamyl alcohol	CC(C)CCO	High	0	0.55	0.538	[6]
Dibutyl phthalate	CCCCOC(=O)C1 = CC=CC=C1C(=O)OCCCC	High	0	0.55	0.602	[6]
Hexanal	CCCCCC=O	High	0	0.55	0.493	[6]
Benzaldehyde	C1 = CC=C(C=C1)C=O	High	0	0.55	0.497	[6]
Nonanal	CCCCCCCCC=O	High	0	0.55	0.499	[6]

**Table 2:** Potential target information filtered by network topology parameters.

Name	BC	CC	DC	EC	LAC	NC
MAPK3	357.3	0.1500	14	0.4222	4.3	11.585
MAPK1	209.3	0.1475	13	0.4092	4.5	11.161
EGFR	160.7	0.1434	12	0.3654	3.8	8.619
ESR1	11.2	0.1390	8	0.3084	4.5	7.074
NR3C1	62.7	0.1379	7	0.2689	3.4	4.300
MAPT	75.5	0.1374	6	0.2110	2.3	3.600
GSK3B	3.0	0.1379	6	0.2669	4.3	5.200
CHRM1	172.0	0.1324	5	0.0220	0.8	2.583
F2	424.0	0.1434	5	0.1210	0.8	1.083
IGF1R	60.0	0.1369	5	0.2061	2.4	3.000
RARA	3.0	0.1358	5	0.1917	2.8	4.167
ADRA1B	116.0	0.1319	4	0.0215	1.0	2.667
PTGS2	168.0	0.1379	4	0.1105	1.0	2.167
ESR2	0.0	0.1358	4	0.2024	3.0	4.000
PRKCA	1.3	0.1358	4	0.1868	2.5	3.333
PTGS1	60.0	0.1250	3	0.0174	0.7	1.500
PARP1	0.0	0.1343	3	0.1533	2.0	3.000
RARB	0.0	0.1343	3	0.1377	2.0	3.000
ADRA1A	0.0	0.1192	2	0.0040	1.0	2.000
ADRA1D	0.0	0.1192	2	0.0040	1.0	2.000
ALOX5	0.0	0.1246	2	0.0172	1.0	2.000
BCHE	60.0	0.1290	2	0.0170	0.0	0.000
CDK1	0.0	0.1290	2	0.0774	1.0	2.000
CHRM3	0.0	0.1196	2	0.0042	1.0	2.000
CHRM5	0.0	0.1196	2	0.0042	1.0	2.000
LDLR	2.0	0.0286	2	0.0000	0.0	0.000
MAOB	0.0	0.1165	1	0.0024	0.0	0.000
CHRM2	0.0	0.1192	1	0.0034	0.0	0.000
DYRK1A	0.0	0.1233	1	0.0283	0.0	0.000
MMP2	0.0	0.1281	1	0.0491	0.0	0.000
HMGCR	0.0	0.0285	1	0.0000	0.0	0.000
HTR2A	0.0	0.0278	1	0.0000	0.0	0.000
SLC6A4	0.0	0.0278	1	0.0000	0.0	0.000
INSR	0.0	0.1229	1	0.0277	0.0	0.000
VLDLR	0.0	0.0285	1	0.0000	0.0	0.000
MPO	0.0	0.1132	1	0.0023	0.0	0.000
NR3C2	0.0	0.1237	1	0.0361	0.0	0.000

**Table 3:** The BE of molecular docking between the bioactive components and the core predicted targets.

Ligand	Proteins	Affinity (Kcal/mol)	Residues	Hydrogen bonds	PI interactions
Benzaldehyde	EGFR	-4.7	MET766, LEU858, PHE856, ASP855, THR854, CYS775, ARG776, LEU777, THR790, ILE789, LEU788, and LYS745	LYS745	PHE856
	ESR1	-5.2	LEU384, LEU387, MET388, LEU391, ARG394, PHE404, GLU353, ALA350, LEU349, and LEU346		
	GSK3B	-5.8	THR291, VAL164, MET227, TYR229, ALA230, MET281, ALA177, LEU156, GLY157, and PHE438	ALA230	
	NR3C2	-5.1	LEU769, LEU772, ALA773, GLN776, ARG817, LEU814, SER811, LUE810, MET807, and PHE829		
	MAPK1	-4.9	VAL39, ILE31, LEU107, ALA52, ASP106, GLN105, LEU106, and CYS166	MET108	
Camphor	EGFR	-4.9	GLY719, SER720, GLY721, ALA722, PHE723, GLY724, THR725, VAL726, LYS745, ASN842, and ASP855	GLY724	
	ESR1	-5.6	LEU525, LEU428, PHE425, ILE424, MET421, LEU391, MET388, LEU387, LEU384, TRP383, LEU346, THR347, PHE404, LEU349, and ALA350		
	GSK3B	-5.2	GLU234, ARG4, VAL164, LYS163, GLY162, GLY159, LYS158, GLY157, LYS179, and ASP292		
	NR3C2	-6.3	MET845, ARG817, LEU814, PHE829, SER811, LEU810, LEU938, MET807, TRP806, LEU769, ASN770, LEU772, ALA773, and GLN776		
	MAPK1	-5.1	VAL39, GLY32, GLU33, GLY34, LYS114, ASP111, LEU156, LYS54, ASN154, SER153, and CYS166		
Carvone	EGFR	-6.1	VAL726, THR854, THR790, GLN791, ALA743, LEU792, MET793, PRO794, LEU844, PHE997, GLY796, CYS797, LEU1001, and LEU718	MET793	
	ESR1	-6.5	LEU428, ILE424, LEU384, LEU387, MET388, LEU391, ARG394, GLU353, ALA350, LEU349, LEU346, and PHE404		
	GSK3B	-6.0	MET281, MET227, GLU228, TYR229, ALA230, THR211, THR291, ASP292, LYS179, ALA177, VAL164, PHE438, and LEU156		
	NR3C2	-6.1	ARG817, LEU814, SER811, LEU810, MET852, MET807, MET845, LEU938, LEU848, LEU769, LEU772, PHE829, ALA773, and GLN776		
	MAPK1	-6.2	ASP111, LEU156, THR116, LYS114, GLU109, MET108, ILE31, LEU107, VAL39, GLN105, CYS166, and ALA54		
DBP	EGFR	-6.4	LEU718, GLY721, PHE723, GLY724, VAL726, EU777, MET766, LEU788, ILE789, THR790, LEU792, ASP855, THR854, LEU747, LYS745, ALA743, ASN842, and LEU844	THR854	
	ESR1	-6.7	GLU353, ALA350, LEU349, THR347, LEU346, ARG394, LEU391, MET388, LEU387, LEU384, TRP383, LEU525, GLY521, MET421, ILE424, PHE425, LEU428, and PHE404		PHE404
	GSK3B	-7.2	THR291, ASP292, ARG4, VAL164, LYS163, GLY162, PHE161, GLY159, LYS158, GLY157, LEU156, LEU181, LYS179, ALA177, GLU234, PHE438, ALA230, TYR229, MET281, GLU228, MET227, and THR211	THR291	
	NR3C2	-6.8	LEU827, LEU769, LEU960, ASN770, TRP806, MET807, LEU772, ALA773, LEU810, GLN776, SER811, LEU814, ARG817, MET845, LEU848, LEU938, PHE941, CYS942, and MET852		PHE829
	MAPK1	-6.0	ASP167, CYS166, GLY169, THR68, GLU71, LEU156, GLN105, TYR64, ILE56, ILE84, VAL39, PRO58, SER153, TYR36, ALA35, LYS151, GLY34, ALA189, and GLY37	LYS54,ARG67	
Estragole	EGFR	-5.4	VAL726, ALA743, LYS745, THR854, THR790, GLN791, LEU792, MET793, LEU844, LEU1001, GLY796, CYS797, LEU718, and PHE997		
	ESR1	-5.7	LEU384, LEU387, MET388, PHE404, LEU391, ARG394, GLU353, ALA350, LEU349, LEU346, MET421, ILE424, PHE425, and LEU428		PHE404
	GSK3B	-6.4	ASP292, THR291, MET281, ALA177, GLU228, TYR229, ALA230, PHE438, VAL164, GLU234, ARG4, LEU156, GLY157, and LYS158		
	NR3C2	-5.6	ARG817, LEU814, SER811, LEU810, MET852, LEU938, CYS849, MET807, LEU848, TRP806, MET845, LEU769, PHE829, LEU72, ALA773, and GLN776		
	MAPK1	-5.6	LEU156, LEU107, ASP106, ILE84, MET108, GLN105, CYS166, GLU109, THR110, ALA52, ASP111, LYS54, VAL39, LYS114, and ILE31		
Geraniol	EGFR	-5.4	THR854, ASP855, LEU788, ILE789, THR790, LEU792, MET793, GLY796, CYS797, LEU718, LEU844, VAL726, ALA743, ILE744, LYS745, and PHE997		
	ESR1	-5.8	MET421, ILE424, PHE425, LEU428, LEU384, LEU387, MET388, LEU391, ARG394, GLU353, ALA350, LEU349, PHE404, THR347, LEU346, LEU525, and MET343	ARG394	
	GSK3B	-6.1	GLU234, ASP292, THR291, MET281, THR211, ALA230, TYR229, GLU228, ALA177, VAL164, LEU156, and PHE438		
	NR3C2	-6.0	LEU814,SER811,LEU810,MET807,TRO806,LEU938,MET845,LEU848,CYS849,LEU769,LEU827,LEU772,MET852,PHE829,ALA773,	GLN776,ARG817	
	MAPK1	-5.5	VAL39, LYS54, ILE53, ILE31, ALA52, GLN105, LEU107, MET108, GLU71, ASP167, LEU156, ILE84, and CYS166	ASP106	
Isoborneol	EGFR	-5.5	THR854, CYS797, GLY796, MET793, LEU792, GLN791, PHE997, LEU844, ARG841, LEU718, GLY719, VAL726, LEU1001, LYS745, and ALA743		
	ESR1	-6.7	LEU384, LEU387, MET388, LEU391, ARG394, LEU525, MET421, PHE425, LEU428, GLU353, ALA350, LEU349, LEU346, MET343, and PHE404		
	GSK3B	-5.8	GLU234, ALA177, LYS179, VAL164, LYS163MET281, PHE161, GLY159, LYS158, GLY157, MET227, THR291, ASP292, and ARG4		
	NR3C2	-6.9	LEU938, MET852, TRP806, MET807, LEU810, SER811, CYS849, LEU848, LEU814, MET845, LEU827, PHE829, ARG817, GLN776, ALA773, LEU72, ASN770, and LEU769		
	MAPK1	-5.8	VAL39, LYS54, ALA52, GLU33, ILE31, GLY34, LYS114, ASP111, GLU109, LEU107, THR110, CYS166, MET108, SER153, ASN154, and LEU156		
Kaempferol	EGFR	-7.0	ARG841, LEU844, THR790, ASP855, THR854, LYS745, ALA743, CYS797, GLY796, LEU718, GLY719, SER720, GLY721, GLY724, THR725, and VAL726	ASP855	
	ESR1	-8.7	MET, ILE424, PHE425, MET343, LEU346, THR347, LEU349, ALA350, PHE404, ARG394, MET388, LEU387, LEU384, TRP383, VAL534, and LEU525		
	GSK3B	-7.9	MET281, THR291, ASP292, VAL164, LYS163, GLY162, GLY159, LYS158, GLY157, LEU156, ARG4, PHE442, PHE438, GLU234, LYS179, ILE180, and LEU181		
	NR3C2	-8.0	LEU814, SER811, LEU810, MET852, LEU938, MET807, PHE941, CYS942, PHE956, THR945, LEU766, LEU769, MET845, ASN770, PHE829, LEU772, ALA773, and GLN776,		
	MAPK1	-8.4	MET108, ASP111, LEU156, ASN154, LYS114, SER153, ILE31, LYS151, GLU33, GLY34, CYS166, ASP167, YS54, GLY37, and VAL39		
Linalool	EGFR	-5.2	LEU844, THR790, LEU792, MET793, GLY796, CYS797, LEU1001, LEU718, PHE997, ALA743, ILE744, LYS745, VAL726, CYS775, THR854, and ASP855		
	ESR1	-5.4	LEU428, PHE425, ILE424, MET421, ARG394, LEU391, MET388, LEU387, LEU384, TRG383, PHE404, GLU353, ALA350, LEU349, and LEU346		
	GSK3B	-5.9	ARG4, THR291, ASP292, MET281, ASN279, GLU278, GLU234, VAL164, ALA177, ALA230, TYR229, LEU156, GLY157, and PHE438	GLU234	
	NR3C2	-5.6	LEU810, SER811, LEU814, MET852, ARG817, LEU848, MET845, LEU938, PHE829, LEU769, LEU772, TRP806, MET807, and ALA773,	GLN776	
	MAPK1	-5.4	VAL39, ILE31, LEU156, ALA52, ILE53, LYS54, ILE103, CYS166, GLN105, ASP106, ALU104, MET108, ILE84, THR110, and ASP111		
Nerol	EGFR	-5.5	LEU718, LEU844, GLY796, MET793, LEU792, GLN791, THR790, ILE789, LEU788, THR854, ASP855, LYS745, ILE744, ALA743, and VAL726		
	ESR1	-5.7	LEU428, PHE425, ILE424, MET421, ARG394, LEU391, MET388, LEU387, LEU384, PHE404, GLU353, ALA350, LEU349, THR347, LEU346, MET343, and LEU525		
	GSK3B	-6.0	LYS179, ALA177, ASP292, THR291, MET281, TYR229, ALA230, GLY157, LEU156, GLU234, PHE438, VAL164, and ARG4	ALA230	
	NR3C2	-6.0	ARG817, LEU814, SER811, LEU810, MET807, TRO806, LEU938, MET852, CYS849, LEU848, LEU827, PHE829, LEU769, LEU772, ALA773, and GLN776		
	MAPK1	-5.5	VAL39, ILE31, GLN105, ASP106, LEU107, MET108, ILE84, ALA52, ILE53, LEU156, LYS54, CYS166, and GLU71	ASP167	
Nerolidol	EGFR	-5.9	CYS775, THR854, ASP855, THR790, LEU792, MET793, LEU718, GLY719, LEU844, LYS745, ILE744, ALA743, and VAL726	ASP855	
	ESR1	-6.4	GLY521, MET421, ILE424, PHE425, LEU428, PHE404, TRP383, LEU384, LEU387, MET388, MEU391, ARG394, GLU353, ALA350, LEU349, THR347, LEU346, MET343, and LEU525		
	GSK3B	-6.8	THR211, ALA177, LYS179, ILE180, LEU181, ASP292, THR291, MET281, VAL164, LYS163, GLY162, PHE161, GLY159, LYS158, GLY157, LEU156, ARG4, GLU234, PHE438, ALA230, GLU228, and MET227		
	NR3C2	-6.4	ASN770, LEU960, LEU772, ALA773, PHE829, GLN776, ARG817, MET852, LEU814, SER811, LEU810, MET845, LEU938, MET807, TRP806, PHE941, CYS942, and LEU766	LEU769	
	MAPK1	-6.5	GLY43, GLU33, GLY32, ILE31, ALA52, ILE84, LYS54, CYS166, ASP167, GLN105, ASP106, LEU156, LEU107, MET108, SER153, THR110, ASP111, VAL39, and LYS114		
Nonanal	EGFR	-4.8	LEU788, ILE789, THR790, LYS745, ILE744, ALA743, VAL726, LEU844, LEU777, MET766, ASP855, and THR854	ASP855	
	ESR1	-4.7	LEU525, LEU428, PHE404, ARG394, LEU391, MET388, LEU387, LEU384, GLU353, ALA350, LEU349, and LEU346		
	GSK3B	-5.1	MET227, GLU228, TYR229, ALA230, ALA177, THR211, PHR438, LEU156, GLY157, VAL164, MET281, ASN279, GLU278, ARG4, GLU234, THR291, and ASP292		
	NR3C2	-4.8	ARG817, LEU814, LEU938, SER811, LEU810, MET807, MET845, LEU769, ASN770, LEU772, PHE829, ALA773, and GLN776		
	MAPK1	-4.4	GLY32, ILE31, LYS114, ASP111, THR110, GLU109, MET108, LEU156, LEU107, ALA52, CYS166, GLN105, LYS54, and CAL39		
Piperitone	EGFR	-6.1	THR854, ASP855, ALA743, LYS745, VAL726, THR790, GLN791, LEU792, MET793, GLY796, and LEU844	MET793	
	ESR1	-6.2	ILE424, PHE425, LEU428, LEU384, LEU387, MET388, LEU391, ARG394, GLU353, ALA350, LEU349, LEU346, and PHE404		
	GSK3B	-6.9	LYS179, ALA177, THR211, MET227, GLU228, TYR229, ALA230, PHE438, LEU156, MET281, and VAL164		
	NR3C2	-6.2	LEU813, SER811, LEU8180, MET852, MET807, MET845, LEU938, LEU769, PHE829, LEU772, ALA773, and GLN776		
	MAPK1	-6.0	VAL39, LYS54, ALA52, ILE31, GLN105, ASP106, LEU107, MET108, LEU156, CYS166, and ASP167		
Quercetin	EGFR	-9.1	LEU844, THR854, ASP855, MET766, LEU777, LEU788, ILE789, THR790, LEU792, MET793, GLY796, CYS797, LEU718, LEU1001, PHE997, VAL726, LYS745, ILE744, and ALA743		LYS745
	ESR1	-8.3	LEU428, TRP383, LEU384, LEU387, MET388, PHE404, LEU391, ARG394, GLU353, ALA350, LEU349, THR347, LEU346, MET343, MET528, LEU525, and VAL534	ARG394	PHE404
	GSK3B	-8.8	ASP292, THR291, THR211, LYS179, ALA177, MET227, GLU228, TYR229, ALA230, PHE438, MET281, LEU156, GLY157, GLY159, VAL164, GLU234, and ARG4	ALA230	
	NR3C2	-5.2	LEU814, SER815, ARG817, MET852, MET845, PHE946, TRH945, PHE956, VAL954, CYS942, PHE941, LEU766, ILE964, LUE960, LEU769, LEU938, ASN770, TRP806, MET807, LEU772, ALA773, LEU810, GLN776, and SER811	PHE829	
	MAPK1	-9.1	ARG67, ILE56, LYS54, ALA52, GLY34, ILE31, LYS114, ASP111, MET108, LEU107, LEU156, CYS166, ASN154, ASP167, SER153, LYS151,VAL39, GLY37, TYR36, and ALA35		
Terpineol	EGFR	-5.9	THR854, ASP855, PHE856, CYS775, ARG776, LEU777, MET766, LEU788, ILE789, THR790, LYS745, ILE744, ALA743, and VAL726		
	ESR1	-6.7	LEU428, LEU384, ILE424, LEU387, MET388, LEU391, ARG394, GLU353, ALA350, LEU349, LEU346, and PHE404	ARG394,GLU353	
	GSK3B	-6.7	ARG4, GLU234, ASP292, THR291, GLU278, MET281, VAL164, LYS158, GLY157, LEU156, PHE438, TYR229, ALA230, and ALA177		
	NR3C2	-6.0	TRP806, GLN776, ALA773, LEU772, ASN770, LEU769, PHE829, LEU810, SER811, LEU814, LEU938, MET852, CYS849, LEU848, and MET845		
	MAPK1	-6.1	VAL39, ILE31, GLY32, GLU33, ALA52, GLN105, ASP106, ILE84, LEU107, MET108, CYS166, LEU156, SER153, and LYS114	ASP111	

## Data Availability

The data used to support the findings of this study are included within the article.

## Abbreviations

OBL: *Ocimum basilicum* L
AD: Alzheimer's disease
DBP: Diterbutyl phthalate
QED: The quantitative estimate of drug-likeness
BC: Betweenness centrality
CC: Closeness centrality
EC: Eigenvector centrality
DC: Degree centrality
LAC: Local average connectivity-based method
NC: Network centrality
BE: Binding energy.
